# Characterization of Neutrophil Subsets in Healthy Human Pregnancies

**DOI:** 10.1371/journal.pone.0085696

**Published:** 2014-02-13

**Authors:** Aloysius Ssemaganda, Lindsay Kindinger, Philip Bergin, Leslie Nielsen, Juliet Mpendo, Ali Ssetaala, Noah Kiwanuka, Markus Munder, Tiong Ghee Teoh, Pascale Kropf, Ingrid Müller

**Affiliations:** 1 Uganda Virus Research Institute - International AIDS Vaccine Initiative, Entebbe, Uganda; 2 Department of Obstetrics and Gynecology, St. Mary’s Hospital, London, United Kingdom; 3 International AIDS Vaccine Initiative Human Immunology Laboratory, Faculty of Medicine, Imperial College London, London, United Kingdom; 4 Section of Immunology, Department of Medicine, Faculty of Medicine, Imperial College London, London, United Kingdom; 5 Third Department of Medicine, Hematology, Oncology and Pneumology, University Medical Center, Mainz, Germany; 6 School of Public Health, College of Health Sciences, Makerere University, Kampala, Uganda; Medical Faculty, Otto-von-Guericke University Magdeburg, Medical Faculty, Germany

## Abstract

We have previously shown that in successful pregnancies increased arginase activity is a mechanism that contributes to the suppression of the maternal immune system. We identified the main type of arginase-expressing cells as a population of activated low-density granulocytes (LDGs) in peripheral blood mononuclear cells and in term placentae. In the present study, we analyzed the phenotype of LDGs and compared it to the phenotype of normal density granulocytes (NDGs) in maternal peripheral blood, placental biopsies and cord blood. Our data reveal that only LDGs but no NDGs could be detected in placental biopsies. Phenotypically, NDGs and LDGs from both maternal and cord blood expressed different levels of maturation, activation and degranulation markers. NDGs from the maternal and cord blood were phenotypically similar, while maternal, cord and placental LDGs showed different expression levels of CD66b. LDGs present in cord blood expressed higher levels of arginase compared to maternal and placental LDGs. In summary, our results show that in maternal and cord blood, two phenotypically different populations of neutrophils can be identified, whereas in term placentae, only activated neutrophils are present.

## Introduction

Transient modulation of innate and adaptive maternal immunity during pregnancy contributes to the creation of an immunosuppressive state allowing implantation and growth of the fetus. Although there is bidirectional communication and migration of fetal and maternal cells throughout pregnancy the paternal antigens expressed by the fetus are not attacked and rejected by the maternal immune system [1]. It is generally accepted that in successful healthy pregnancies multiple mechanisms provided by both the mother and the fetus contribute to the development and maintenance of immune tolerance and immune privilege [2]. In normal pregnancies there is an increased systemic inflammation, enhanced number of polymorphonuclear cells (PMN), a low Th1/Th2 balance, a decrease in peripheral NK cells, and an increased number of regulatory T cells [3], [4], [5]. Although major progress has been made in the understanding of immune mechanisms that prevent rejection of the fetus, the generation of this immunosuppressive state is not fully elucidated [6].

Indoleamine 2,3 dioxygenase (IDO), a tryptophan-catabolizing enzyme expressed by both the maternal decidua and fetal trophoblast and catabolites of the tryptophan metabolism such as kynurenine and picolinic acid can inhibit lymphocyte activation and have been shown to contribute to the Th2 bias and to tolerance induction and maintenance in pregnancy [7].

We previously showed that another amino acid catabolizing enzyme, arginase, is upregulated in term placentae as well as in the peripheral blood at parturition and contributes to suppression of maternal immune responses in healthy pregnancies [8]. Two isoforms of arginase exist, arginase I and II, which both hydrolyze the same substrate, the amino acid L-arginine, to ornithine and urea. They differ in cellular and subcellular expression and regulation. Both arginase isoforms are expressed in the human placenta [9] and increased enzymatic activity of arginase results in elevated substrate consumption and decreased L-arginine in the extracellular fluid. L-arginine is essential for T cell activation and the generation of nitric oxide (NO) [10], [11]. Indeed, reduction of extracellular L-arginine by the enzymatic activity of arginase impairs maternal T cell responses; thus, arginase-induced L-arginine deprivation is one of the pathways ensuring T cell hyporesponsiveness and immune privilege at the feto-maternal interphase [8]. We have previously identified the phenotype of arginase-expressing cells in maternal blood and placentae as low-density granulocytes (LDGs) that co-purify with PBMCs following density gradient centrifugation. This difference in density distinguishes this population from the remaining granulocytes that co-purify with the erythrocyte fraction following density gradient centrifugation and thus have been named normal-density granulocytes (NDGs).

In the present study we analyzed the phenotype and frequency of normal and low density granulocytes obtained from maternal peripheral blood, placental biopsies and cord blood.

## Materials and Methods

### Ethics Statement

This study protocol was approved by the NHS NRES Committee South Central Oxford A (REC reference 12/SC/0721; IRAS project ID 1181020).

### Subjects and Samples

All individuals gave written, informed consent before participation. Pregnant women (n = 7; mean age 34years, mean BMI 26) were recruited at the time of elective caesarean section; the mean gestational age at delivery was 38 weeks and 6 days. Exclusion criteria included any major complication of pregnancy or intercurrent illness, such as pre-eclampsia, pre- or post-term labor (<37 weeks or >42 weeks), intra-uterine growth retardation, viral, bacterial or parasitic infections.

Ten ml of maternal peripheral blood and of cord blood were collected in EDTA tubes and PBMCs were isolated by density gradient centrifugation on Histopaque 1077 (Sigma). Neutrophils were isolated from the erythrocyte fraction by dextran sulphate sedimentation [12].

Placentae were harvested directly after parturition and up to five small biopsies were taken through the full thickness of the placenta. Single cell suspensions were obtained by homogenizing biopsies in PBS on cell dissociation sieves and purified from debris by Histopaque 1077 density gradient centrifugation. The placental cells (PlaC) obtained were washed and resuspended in PBS. All experiments were performed on fresh cells, immediately after processing.

### Flow Cytometry

The following antibodies purchased from Biolegend were used: CD66b^FITC^ (involved in respiratory burst, adhesion molecule, present in the membrane of specific granules); CD15^PE^ (involved in cell-cell interactions, phagocytosis, stimulation of degranulation, and respiratory burst); CD63^PE-Cy7^ (marker for release of azurophilic granules); CD33^PE-Cy5^ (marker of immature neutrophils, adhesion molecule); CD16^AF700^ (Marker of mature granulocytes, involved in degranulation). In addition, Zenon Pacific blue conjugated Arginase-1 (Hycult Biotechnology/Invitrogen) was used to detect intracellular arginase 1. The Live/Dead Fixable Near-IR dye (Invitrogen) was used to distinguish live and dead cells.

Cells isolated from cord blood, PlaCs and PBMC were washed and incubated with 20 µl FcR blocking reagent for 5 minutes and stained for 20 minutes with cell surface markers at room temperature. Cells were washed with FACS medium and fixed with cold 4% formaldehyde on ice for 20 minutes.

For intracellular staining, 0.5% saponin was used to permeabilize the cells for 20 minutes at room temperature and then stained with arginase 1 Pacific blue conjugated antibody for a further 20 minutes. The cells were washed and analyzed immediately with an LSRII flow cytometer (BD Bioscience). The results were analyzed using FlowJo v9.6.2 (Tree Star, Ashland, OR).

### Statistical Analyses

Data were evaluated for statistical differences using a two-tailed Mann-Whitney test and a Kruskal-Wallis test when appropriate (GraphPad PRISM version 5); differences were considered significant at p<0.05. Results are expressed as median ± SEM.

## Results

### Phenotype and Frequency of Neutrophil Subpopulations in Maternal Blood, Cord Blood and Term Placentae

We have previously shown that the cells expressing arginase in the PBMCs and placentae of pregnant women are a population of low-density granulocytes (LDGs) that co-purify with PBMCs following density gradient centrifugation [8], [13]. This difference in density distinguishes this population from the remaining granulocytes that co-purify with the erythocyte fraction following density gradient centrifugation and thus have been named normal-density granulocytes (NDGs).

Here we first determined whether both neutrophil subpopulations, LDGs and NDGs, were detectable in the maternal and cord blood and in the term placentae. Whereas both LDGs and NDGs were isolated from maternal and cord blood, NDGs could not be isolated from term placentae despite using different methods such as Histopaque 1119 density gradient centrifugation or isolation by dextran sedimentation from the erythrocyte fraction of Histopaque 1077 gradients.

**Figure 1:**
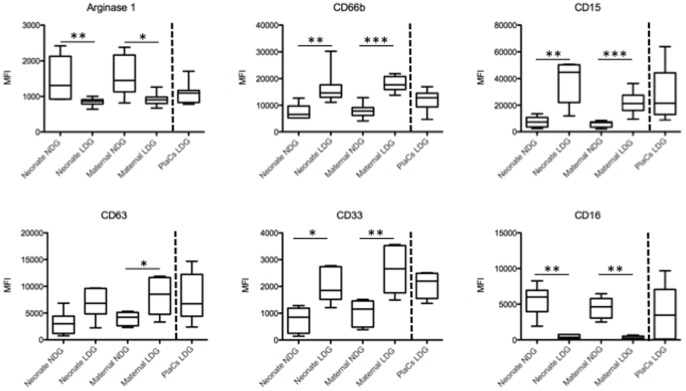
Phenotypic analysis of LDGs and NDGs. LDGs and NDGs were isolated as described in materials and methods (n = 7) and the expression levels of arginase, CD66b, CD15, CD63, CD33 and CD16 were determined by flow cytometry. Statistical significance was determined by a two-tailed Mann-Whitney test. Box = interquartile range and median; whiskers = range.

First we compared the expression levels of arginase, activation and maturation markers by LDGs and NDGs in maternal and cord blood since we had previously shown that the expression levels of arginase, CD15, CD16, CD33, CD63 and CD66b are modulated on LDGs [12]. The expression levels on placental LDGs is shown throughout Figure 1 (after the broken line) for comparison. As shown in Figure 1 and Tables 1 and 2, NDGs present in both maternal and cord blood express significantly more arginase than LDGs; the reduced expression of CD63 on NDGs is in agreement with the higher arginase levels and indicate that these cells are less activated and have not degranulated arginase-containing CD63+ azurophilic granules. The lower arginase expression by LDGs in cord blood, maternal blood and in the placenta corresponds with increased CD63 expression and indicate that these cells had been activated and had degranulated arginase+ azurophilic granules. Furthermore, the increased expression of CD66b by LDGs from all three sources also indicates that LDGs are more activated than NDGs.

**Table 1:** Expression levels of arginase and phenotypic markers of NDGs and LDGs in cord blood.

CORD BLOOD	NDGs	LDGs	*p* values
**Arginase 1**	1312±249	860±42	**0.0047**
**CD66b**	6588±1153	14605±2414	**0.0023**
**CD15**	10896±1664	44720±6046	**0.0023**
**CD63**	3004±880	6820±1017	0.0513
**CD33**	851±216	1851±296	**0.0159**
**CD16**	6006±871	364±136	**0.0043**

NDGs and LDGs were isolated from cord blood (n = 7) as described in materials and methods. Expression levels (MFI) of phenotypic markers were determined by flow cytometry. Statistical significance was determined by a two-tailed Mann-Whitney test.

**Table 2:** Expression levels of arginase and phenotypic markers of NDGs and LDGs in maternal peripheral blood.

MATERNAL BLOOD	NDGs	LDGs	*p* values
**Arginase 1**	1452±216	906±69	**0.0262**
**CD66b**	7836±1021	17682±1072	**0.0006**
**CD15**	6820±914	21207±3198	**0.0006**
**CD63**	4227±505	8515±1259	**0.0350**
**CD33**	1154±198	2658±378	**0.0043**
**CD16**	4637±531	247±86	**0.0012**

NDGs and LDGs were isolated from maternal blood (n = 7) as described in materials and methods. Expression levels (MFI) of phenotypic markers were determined by flow cytometry. Statistical significance was determined by a two-tailed Mann-Whitney test.

The expression levels CD33, a marker of immature neutrophils, were significantly higher in LDGs present in placentae, maternal and cord blood and indicate that LDGs might be a heterogenous population containing both mature and immature neutrophils. CD15 and CD16 are expressed by mature neutrophils and the increased expression of CD15 on LDGs from the three compartments analyzed could be due to upregulation of this molecule in response to activation and degranulation [14].

In conclusion, these results show that LDGs are phenotypically different from NDGs in both maternal and cord blood.

**Figure 2:**
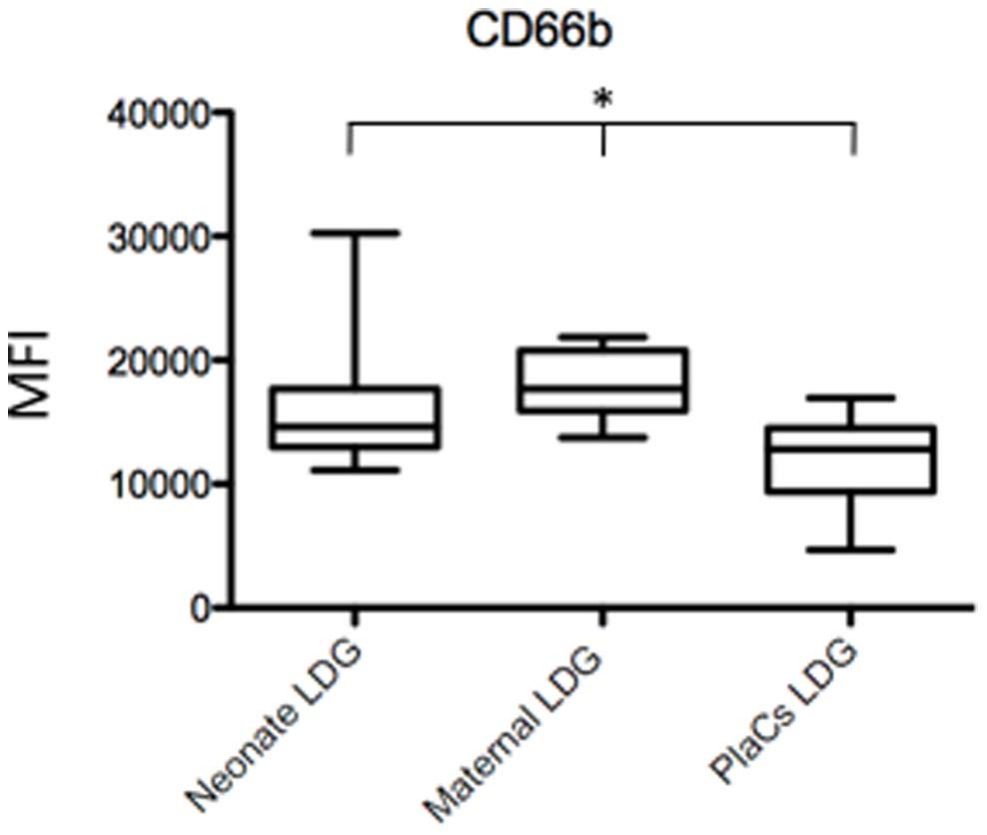
Comparison of the phenotype of LDGs in neonate and maternal blood and placentae. LDGs were isolated as described in materials and methods (n = 7) and the expression levels of CD66b was determined by flow cytometry. Statistical significance was determined by a kruskal-Wallis test. Box = interquartile range and median; whiskers = range.

Next, we determined whether there are phenotypic differences in LDGs from the three compartments. As shown in Figure 1 and Table 3, the phenotype of LDGs in placentae, maternal and cord blood was similar, except for the expression levels of CD66b (Figure 2), which was lower in the placentae, as compared to maternal and cord blood. Results in Table 4 show that the phenotype of NDGs is similar in maternal and cord blood; however, NDG could not be isolated from placentae.

**Table 3:** Comparison of expression levels of arginase and phenotypic markers of LDGs in placentae, cord and maternal blood.

LDGs	Cord blood	Maternal blood	Placentae	*p* values
**Arginase 1**	860±42	906±69	1097±118	0.2801
**CD66b**	14605±2414	17682±1072	12818±1499	**0.0233**
**CD15**	44720±6046	21207±3198	21474±7353	0.1961
**CD63**	6820±1017	8515±1259	6738±1686	0.6977
**CD33**	1851±296	2658±378	2199±220	0.4565
**CD16**	364±136	247±86	3472±1438	0.2368

LDGs were isolated from maternal and cord blood (n = 7) as described in materials and methods. Expression levels (MFI) of phenotypic markers were determined by flow cytometry. Statistical significance was determined by a two-tailed Mann-Whitney test.

**Table 4:** Comparison of expression levels of arginase and phenotypic markers of NDGs in placentae, cord and maternal blood.

NDGs	Cord blood	Maternalblood	Placentae	*p* values
**Arginase 1**	1312±249	1452±216	None recovered	0.9452
**CD66b**	6588±1153	7836±1021	None recovered	0.6282
**CD15**	10896±1664	6820±914	None recovered	0.5338
**CD63**	3004±880	4227±505	None recovered	0.3095
**CD33**	851±216	1154±198	None recovered	0.2468
**CD16**	6006±871	4637±531	None recovered	0.2525

NDGs were isolated from maternal and cord blood (n = 7) as described in materials and methods. Expression levels (MFI) of phenotypic markers were determined by flow cytometry. Statistical significance was determined by a two-tailed Mann-Whitney test.

**Figure 3:**
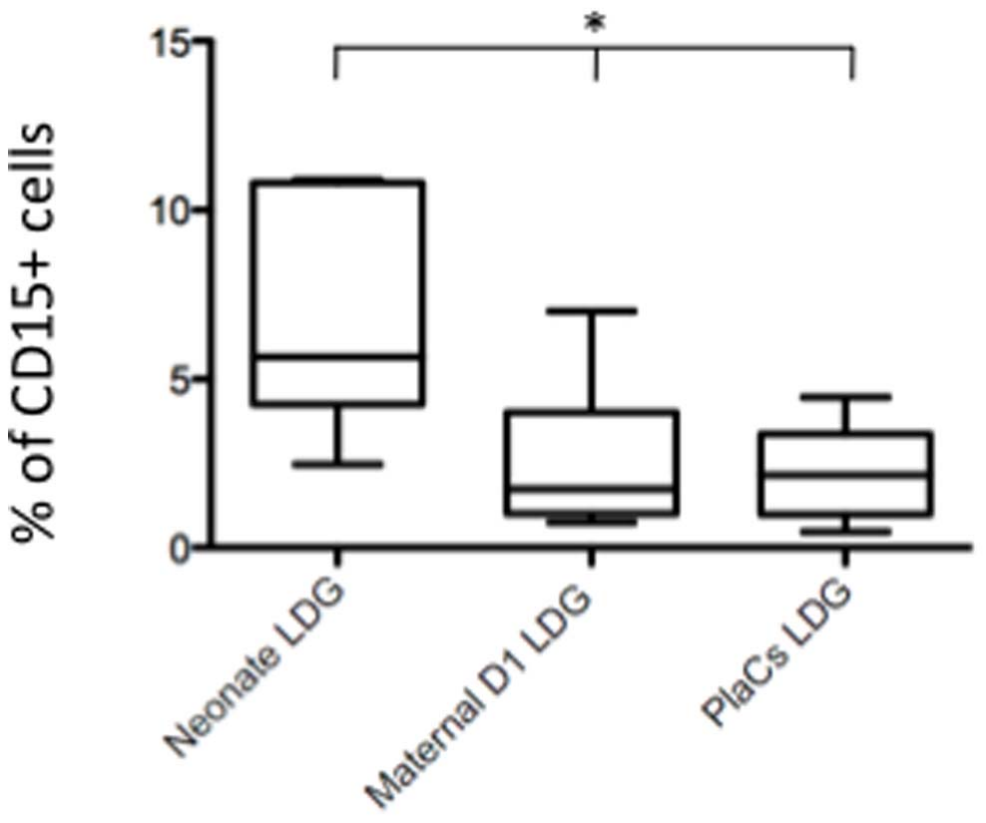
Percentage of LDGs in neonatal and maternal blood and placentae. LDGs were isolated as described in materials and methods (n = 7) and the percentage of CD15^+^ arginase^+^ cells was determined by flow cytometry. The percentage of LDGs present in the peripheral blood of healthy controls was 0.24±0.3 [12] Statistical significance was determined by a kruskal-Wallis test. Box = interquartile range and median; whiskers = range.

Finally, we determined the frequency of LDGs and our results show that there are significantly more LDGs in cord blood as compared to the placentae or maternal blood (Figure 3 and Table 5).

**Table 5:** Percent of Arginase1^+^CD15^+^ LDGs present in placentae, cord blood and maternal blood.

Arginase1^+^CD15^+^LDGs	Cord blood	Maternalblood	placentae	*p* valueip
**Arginase 1**	5.66±1.24	1.723±0.95	2.14±0.53	0.0237

LDGs were isolated from maternal and cord blood and placentae (n = 7) as described in materials and methods. The percentage of CD15^+^ arginase^+^ cells was determined by flow cytometry. Statistical significance was determined by a Kruskal-Wallis test.

## Discussion

Modulation of both innate and adaptive immune responses is crucial in successful pregnancy since the symbiosis between mother and fetus is not due to immunological ignorance but rather to suppression of maternal immune responses. Arginase-mediated L-arginine catabolism is a well-established mechanism of T cell suppression [10], [15], [16]. We showed previously that arginase activity is significantly increased in the placenta and in maternal blood and that arginase-induced L-arginine catabolism is one of the pathways contributing to suppression of maternal T cells responses in healthy human pregnancies [8]. Furthermore, we demonstrated recently that arginase activity and L-arginine levels return to physiological levels after birth [17].

In the present study we further characterized the phenotype of LDGs and compared it to normal density granulocytes (NDGs) isolated from placental biopsies, maternal and cord blood. At parturition both subsets of neutrophils are present in peripheral maternal blood and in cord blood, however, from term placentae only LDGs could be isolated and despite using different methods it was not possible to isolate normal density granulocytes from term placentae. After density gradient centrifugation of blood, NDGs sediment with erythrocytes whereas LDGs co-purify with the PBMC fraction suggesting that their density is lower [13]. They also differ phenotypically: NDGs from maternal and cord blood express significantly higher cell surface levels of CD16 and significantly more intracellular arginase as compared to LDGs. The cell surface levels of CD63 were significantly lower in NDGs than in LDGs. In human neutrophils arginase is contained in azurophilic [18] and gelatinous granules and upregulation of CD63 coincides with the release of azurophilic granules [19]. Thus, our results showing that CD63 is increased in LDGs suggest that these cells have been activated and have degranulated. Furthermore, increased expression levels of CD15 have also been associated with degranulation [14]. Indeed, the increased cell surface expression of CD66b, CD15, and CD63 on LDGs support this conclusion.

The frequency of LDGs was highest in cord blood, however, the phenotype of LDGs in maternal blood, term placentae and cord blood was similar and the phenotype of NDGs in maternal and cord blood was also not significantly different.

CD15^+^ neutrophils in the PBMC fraction have not only been observed in pregnancy (present study and [8]. It was also reported in a range of conditions including HIV [12], visceral leishmaniasis [20], cancer [15], [16], [21], trauma [22], and systemic lupus erythromatosus [23]. All of these conditions are frequently accompanied by various degrees of immune suppression.

Pregnancy has been associated with a shift in Th1/Th2 balance, reduced type 1responsiveness [3], [4] and enhanced innate immunity. Indeed, the number of neutrophils increases in pregnancy and these cells undergo functional and metabolic changes [24], [25], [26]. Delayed apoptosis of neutrophils in normal pregnancy promotes inflammation to persist and may contribute to pregnancy-associated neutrophilia and pregnancy-induced inflammatory changes in neutrophils in the peripheral blood are akin to those of sepsis [27]. The function of neutrophils in pregnancy has to be very tightly controlled since enhanced inflammatory responses have been linked to pregnancy complications such as preeclampsia.

Neutrophils home to inflammatory sites, they can phagocytose pathogens, degranulate and release web-like structures, the neutrophil extracellular traps (NETs) that are implicated in inflammation and immunity [28]. Placental micro-debris has been shown to activate neutrophils and induce the formation of NETs in a dose-dependent manner [29]. NETs have been suggested to contribute to placental hypoxia in patients with preeclampsia [30]; however, the interplay between micro-debris, neutrophil activation and NETs in successful human pregnancies remains to be determined.

Arginase-mediated L-arginine catabolism is not only impacting on maternal immune responses [8]; amino acids such as L-arginine are also important for the developing fetus. Fetal growth is critically dependent on placental nutrient transport; specialized transporters can pass them to the fetus [31]. Placental amino-acid uptake takes place on the microvillous membrane of the syncytiotrophoblast and the efflux to the fetus is mediated by transporters on the basal membrane. The mammalian target of rapamycin (mTOR) pathway in the placenta regulates amino acid transporters such as L-arginine, and it has been proposed that the placental mTOR pathway constitutes a link between maternal nutrients and fetal growth [32].

The signals resulting in activation, degranulation of neutrophils and their development into LDGs in pregnancy remain to be determined and further work is required to identify the pathways leading to the generation of LDGs.
